# Allograft Reconstruction for the Treatment of Musculoskeletal Tumors of the Upper Extremity

**DOI:** 10.1155/2013/925413

**Published:** 2013-02-14

**Authors:** Luis A. Aponte-Tinao, Miguel A. Ayerza, D. Luis Muscolo, German L. Farfalli

**Affiliations:** Institute of Orthopedics “Carlos E. Ottolenghi,” Italian Hospital of Buenos Aires, 1199 Buenos Aires, Argentina

## Abstract

In comparison with the lower extremity, there is relatively paucity literature reporting survival and clinical results of allograft reconstructions after excision of a bone tumor of the upper extremity. We analyze the survival of allograft reconstructions in the upper extremity and analyze the final functional score according to anatomical site and type of reconstruction. A consecutive series of 70 allograft reconstruction in the upper limb with a mean followup of 5 years was analyzed, 38 osteoarticular allografts, 24 allograft-prosthetic composites, and 8 intercalary allografts. Kaplan-Meier survival analysis of the allografts was performed, with implant revision for any cause and amputation used as the end points. The function evaluation was performed using MSTS functional score. Sixteen patients (23%) had revision surgery for 5 factures, 2 infections, 5 allograft resorptions, and 2 local recurrences. Allograft survival at five years was 79% and 69% at ten years. In the group of patients treated with an osteoarticular allograft the articular surface survival was 90% at five years and 54% at ten years. The limb salvage rate was 98% at five and 10 years. We conclude that articular deterioration and fracture were the most frequent mode of failure in proximal humeral osteoarticular reconstructions and allograft resorption in elbow reconstructions. The best functional score was observed in the intercalary humeral allograft.

## 1. Introduction

Excisions of a bone tumor in the upper extremity may result in a large residual osseous defect and the loss of periarticular soft-tissue stabilizers of the shoulder [1–10], elbow [11, 12], or wrist [13–15] with potentially deleterious effects on both function and viability of the limb. For these locations, there are different reconstructions options including prosthetic devices [3, 5–7], biological constructs either with autografts [5, 6] or allografts [1–15], or the combination of allograft with prosthesis [7–11].

Reconstruction with a massive allograft is preferred in our service due to the possibility of obtaining supporting mechanical loads and the ability to attach host ligaments and muscles to the grafts.

The purpose of this study was to investigate the survival of allograft reconstructions in the medium to long term, to determine factors associated with their failure, and to analyze the final functional score compared to the anatomical site and the type of the reconstruction.

## 2. Patients and Methods

From January 1990 to December 2008, we performed a consecutive series of  72 patients with a musculoskeletal tumor from the upper limb who underwent reconstruction with a massive allograft. Two patients were excluded due to a lack of adequate followup data, leaving 70 cases for analysis. 

**Figure 1:**
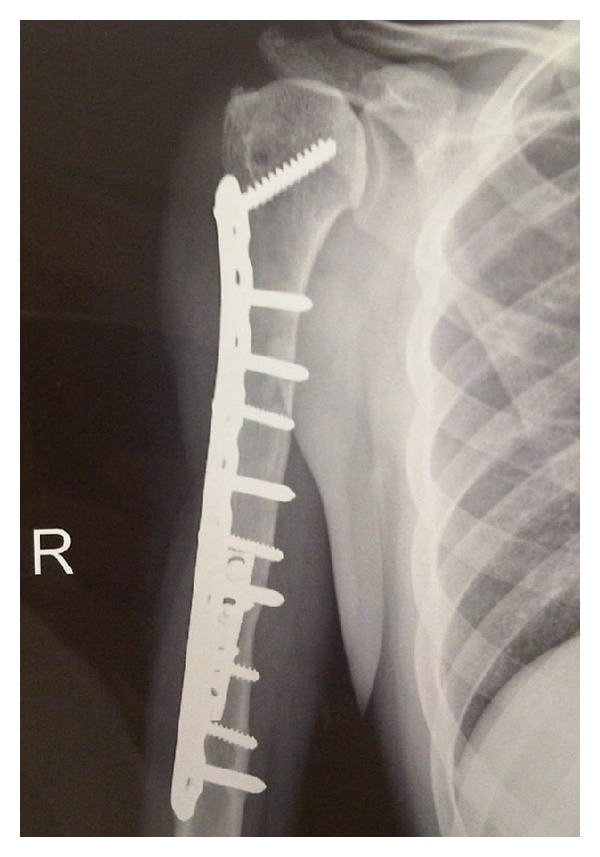
Anteroposterior radiograph of an osteoarticular allograft of the proximal humerus after 5 years of reconstruction.

**Figure 2:**
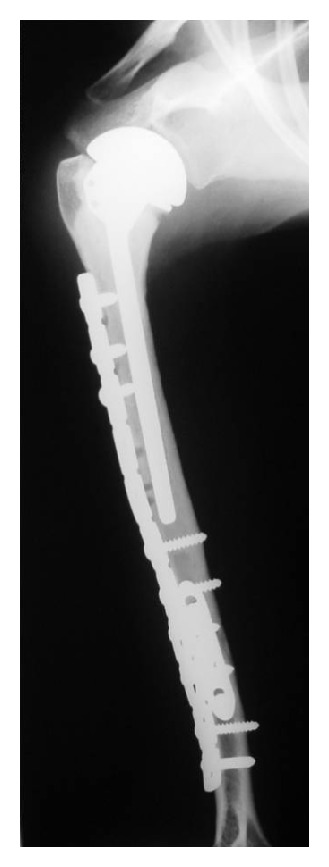
Anteroposterior radiograph of an APC of the proximal humerus showing adequate union of the junction.

**Figure 3:**
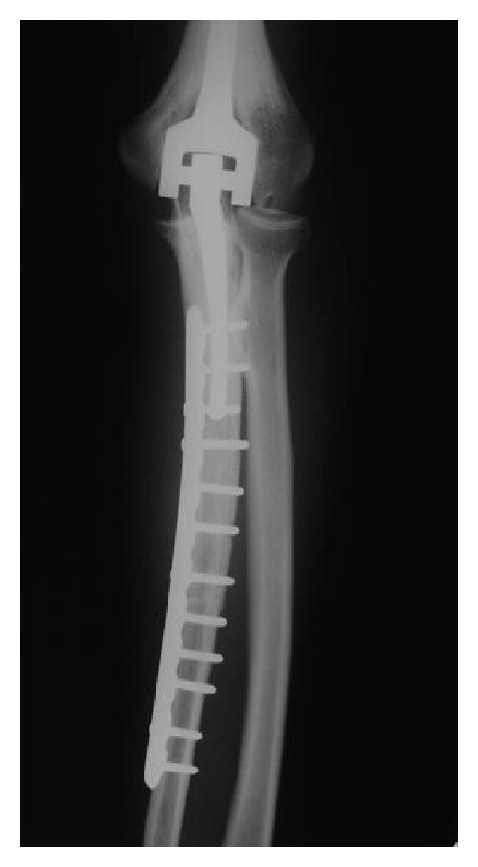
Anteroposterior radiograph of an APC of the elbow after resection of the proximal ulna.

**Figure 4:**
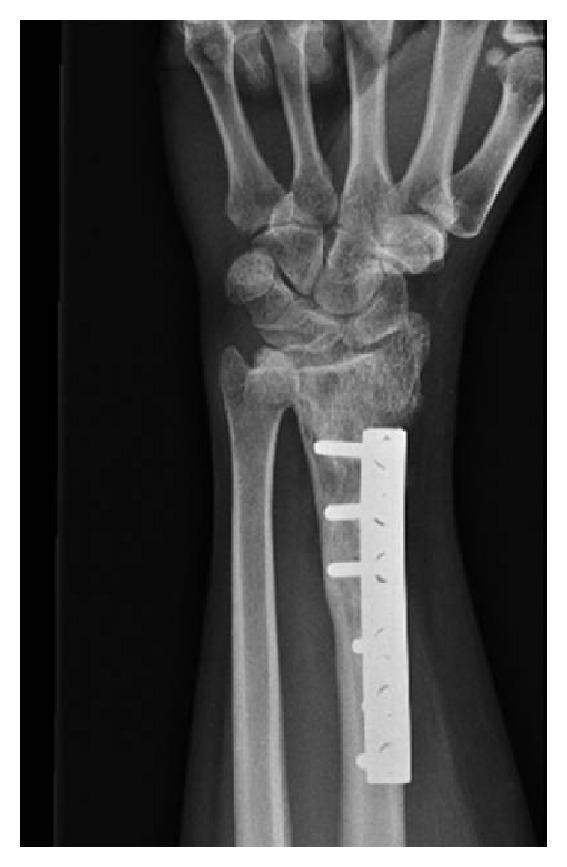
Anteroposterior radiograph 16 years after distal radius osteoarticular reconstruction. Although degenerative changes are evident, the patient is asymptomatic with excellent function.

Of the 70 reconstructions, 38 were osteoarticular allografts, 23 were allograft-prosthetic composites (APC), and 9 were humeral intercalary allografts. Of the 38 osteoarticular reconstructions, 21 were of the proximal humerus (Figure 1), 16 were of the distal radius (Figure 4), and one of the distal humerus. Of the 23 allograft-prosthetic composites, 16 were proximal humeral reconstructions (Figure 2), and 7 were elbow reconstructions (Figure 3). 

Demographic data, diagnosis, site of the neoplasm, operations performed, surgical complications, outcomes after surgery, date of last followup evaluation, and local recurrence were reviewed for all patients.

There were 38 men and 32 women in the study group. The mean age at presentation was 32 years (range 4–71 years). The most common indication for reconstruction was chondrosarcoma in 18 patients, followed by osteosarcoma in 15, giant cell tumors in 15, metastasis in 6, Ewing sarcoma in 5, chondroblastoma in 2, and others types of tumors in the remaining 9 patients. The mean duration of followup was 5 years for patients who survived the original disease (range 1–20 years).

Postoperatively, patients were seen at 1 week, 2 weeks, 1 month, 2 months, 3 months, and then every 3 months thereafter until 2 years, after which we met annually. Beginning 1 month after the operation, we obtained plain radiographs at every visit. We performed functional evaluation using the revised 30-point functional classification system established by the MSTS [16], which assessed pain, function, emotional acceptance, hand positioning, dexterity, and lifting ability. Each variable was assessed on a 5-point scale. Function was compared according the anatomical site and the type of reconstruction performed. Surgical complications were defined according to the Clavien-Dindo classification [17] that separates complications in five grades: Grade I, any deviation from the normal postoperative course without the need for pharmacologic treatment or surgical, endoscopic, and radiographic interventions, with acceptable therapeutic regimens including drugs, such as antiemetics, antipyretics, analgesics, diuretics, electrolytes, and physiotherapy; Grade II, complication requiring pharmacologic treatment with drugs other than those allowed for Grade I complications; Grade III, complication requiring surgical, endoscopic, or radiographic intervention; Grade IV, life-threatening complication; and Grade V, death of a patient. We analyzed only Grades III, IV, and V complications in this series.

We considered an allograft to have failed when it was removed through either a revision procedure or an amputation, and in osteoarticular reconstructions, we considered a joint to have failed when the allograft was not removed, but symptomatic degeneration of the joint was present at the last followup.

The rates of survival of the allograft, the limb, and the joint surface were estimated with the use of the Kaplan-Meier method, starting on the date of the operation and ending on the date of removal, amputation, or the latest followup. Cox regression analysis was done to determine whether age, gender, diagnosis, type, and site of the reconstructions were independent prognostic factors. The log-rank test was used to compare the survivorship curves. A *P* value of <0.05 was considered to be significant.

## 3. Results

**Figure 5:**
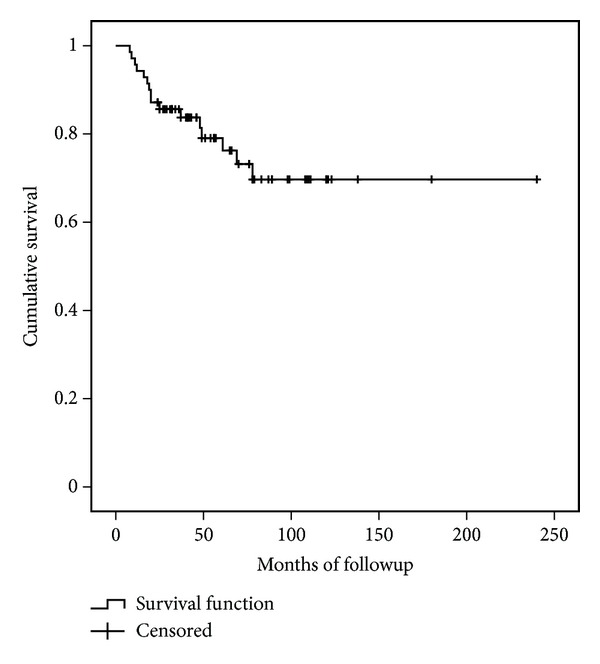
Allograft survival.

Allograft survival (Figure 5) at five years was 79% (CI95%: 68%–90%) and 69% (CI95%: 55%–83%) at ten years for failure from any cause as the end point (Figure 1). The limb survival rate was 98% at five and 10 years (CI95%: 94%–100%).

We identified 22 patients with complications requiring a second surgery (32%), including 7 local recurrences, two deep infections, 5 fractures, 5 resorptions, and 3 nonunions. However, only in 16 patients (23%) the allograft was removed (4 local recurrences, 5 resorptions, 2 infections, and 5 fractures) (Table 1). In 6 patients the allograft was not removed (3 local recurrences in soft tissue and the 3 nonunions).

Seven patients had local recurrences. Three recurrences were in the soft tissue and were resected with wide margins; in these three cases the reconstructions were not revised, so the allograft reconstruction was not affected. In four patients the allograft was compromised by the local recurrence. In these four cases the graft was removed with the local recurrence and only two of them were reconstructed. One was reconstructed with a new allograft (distal radius) and the other with a proximal humerus endoprosthesis. The remaining two patients were treated with a resection arthroplasty and with an amputation (both of them located in the humerus). 

Two patients had an acute deep infection, in which the allograft was removed, and a temporary cement spacer with antibiotics was implanted. After 6 weeks of intravenous antibiotics and another 6 weeks of oral antibiotics, we reimplanted another allograft in one patient (wrist arthrodesis), and the other patient was reconstructed with proximal humeral prosthesis. 

Five patients suffer an allograft fracture, and all occurred in proximal humeral osteoarticular reconstructions. All patients required a second operation, including a second allograft reconstruction with an APC in 3 patients, a second osteoarticular allograft in one, and a cement spacer in the remaining patient. 

Five patients had allograft resorptions, all of them occurred after an elbow reconstruction (four APCs and one osteoarticular allograft). Of the failed elbow reconstructions, two were converted to an elbow endoprosthesis, two had a resection arthroplasty, and one had a cement spacer.

The three patients who underwent nonunion were treated with autologous bone graft and a new plate, without revision of the reconstruction. 

**Figure 6:**
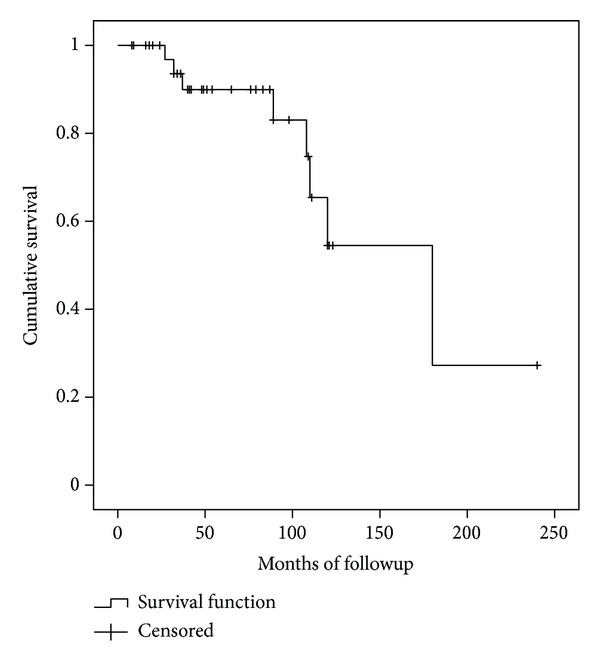
Articular surface survival.

The articular surface survival (Figure 6) of the group of patients treated with an osteoarticular allograft was 90% (CI95%: 79%–100%) at five years and 54% (CI95%: 39%–69%) at ten years (Figure 2). All symptomatic articular deteriorations occurred in the proximal humeral reconstructions, and none of them required revision because of this event.

The only independent prognostic factors that were found to be significant on Cox regression analysis, with revision for any cause as the end point, were the gender of the patient (more frequent in males: *P* = 0.02).

 For the patients who retained the reconstruction (54 cases), the mean MSTS functional score at last followup was 26 of 30 (83%, range 18–30). The best mean functional score was observed in the intercalary humeral allograft group. (mean 30: 100%). The worst functional score was observed in proximal humeral osteoarticular allograft group (23 points, range 18–26), and this lower score was mainly related with patients who had a significant articular deterioration (Table 2). 

## 4. Discussion

In comparison with the lower extremity, there is relatively paucity literature reporting survival and clinical results of allograft reconstructions after excision of a bone tumor of the upper extremity. We include in this report all reconstructions done in the upper extremity done in our unit. 

There are some limitations to this study. This is a retrospective study with a relatively low number of patients and followup. In addition, there are many variables related to the anatomic location of the reconstructions. Despite these limitations, we believe that this series is one of the largest series reported in the literature, and our results may provide some trends in the treatment of massive bone defects in the upper limb.

Regarding anatomical site, most publications are related to the proximal humerus. Osteoarticular allografts are used less frequently than in the lower extremity, but there are reports regarding this type of reconstruction in the proximal humerus. Although some authors reported satisfactory results with osteoarticular allografts of the proximal humerus [1] and survival rates of 78% at five years [2], recent reports suggest that better or at least similar results are obtained with allograft prosthesis composite and endoprosthesis reconstructions regarding reconstruction survival and complications [3–8]. Peabody [4] report that due to functional limitations as well as an extremely high rate of complications, they do not use osteoarticular allografts to replace the proximal aspect of the humerus. However, in a recent report [7] that analyzed 38 reconstructions of the proximal humerus the endoprosthetic group presented the smallest complication rate of 21%, compared to 40% in the allograft prosthesis composite and 62% in the osteoarticular allograft group. However, in another report that analyzed 45 patients [5] reconstructed after tumor resection of the proximal humerus they found that all limb-salvage procedures for the proximal humerus were satisfactory for long-term survival, but none of the 26 disease-free surviving patients was able to abduct their shoulder more than 90°, and only five could achieve active abduction of more than 30°. The survival rate was 83% for endoprosthesis, 79% in clavicula prohumero, and 75% in osteoarticular allograft [5]. 

Reconstructions with APC in the proximal humerus avoid problems of endoprosthesis or osteoarticular allografts used alone [8–10]. In our series the higher amount of fractures occurred at shoulder reconstructions with osteoarticular allografts, and these complications could be avoided with an APC. In recent reports [8, 10] there are not differences regarding complications or survival with other methods.

Although, reports on elbow reconstructions [11, 12] showed satisfactory functional outcome and survival, both reports included trauma and tumor patients. In our series, we found high complication rate (75%) and a mean functional score of 24 points. Five of seven patients' present allograft resorption, and this complication was noted in previous report [12].

All distal radius reconstructions in this series were osteoarticular allografts. In our series we found low complication rate (19%) and high functional score (28 points). Similar results are found in the literature [13–15]; however, all series include a high percent of patients with benign tumors (GCT). This could lead to less damage of soft-tissue structures and better survival of the patient and reconstruction. Although degenerative changes are reported [14], these are usually asymptomatic (Figure 4).

The lower complication rate and the best mean functional score were observed in the intercalary humerus allograft group. Van Isacker et al. [18] report in a series of forearm allograft similar results, they found that intercalary allograft had fewer complications than osteoarticular allografts, and they had a better functional MSTS score.

## 5. Summary

This study showed that allograft reconstruction after a tumor resection of the upper limb may be durable, with a 69% survival rate at ten years. Despite the 32% incidence of complications, only 16 patients (23%) required an allograft removal and were considered as failures. We conclude that articular deterioration and fracture were the most frequent mode of failure in shoulder reconstructions and allograft resorption in elbow reconstructions. The humeral intercalary allografts had the lesser complication rate and the best functional score. 

## Figures and Tables

**Table 1:** Allograft complications according the different types of reconstructions.

Reconstruction	Local recurrence	Infection	Fracture	Resorption	Nonunion	Total (%)
PHOA	2	—	5	—	—	33%
PHAPC	1	1	—	—	2	25%
HIA	1	—	—	—	—	11%
ER	1	—	—	5	—	75%
DROA	2	1	—	—	1	25%

PHOA: proximal humerus osteoarticular allograft; PHAPC: proximal humerus allograft prosthetic composite, HIA: humeral intercalary allograft; ER: elbow reconstructions; DROA: distal radius osteoarticular allograft.

**Table 2:** Mean MSTS functional results comparison of different types of reconstructions.

Reconstruction	Pain	Function	Emotional acceptance	Hand positioning	Dexterity	Lifting ability	Total
PHOA	4	3	4	3	5	4	23
PHAPC	4	4	5	3	5	4	25
HIA	5	5	5	5	5	5	30
ER	3	4	4	4	5	4	24
DROA	4	4	5	5	5	5	28

PHOA: proximal humerus osteoarticular allograft; PHAPC: proximal humerus allograft prosthetic composite, HIA: humeral intercalary allograft; ER: elbow reconstructions; DROA: distal radius osteoarticular allograft.

## References

[1] Gebhardt MC, Roth YF, Mankin HJ (1990). Osteoarticular allografts for reconstruction in the proximal part of the humerus after excision of a musculoskeletal tumor. *Journal of Bone and Joint Surgery—Series A*.

[2] DeGroot H, Donati D, Di Liddo MD, Gozzi E, Mercuri M (2004). The use of cement in osteoarticular allografts for proximal humeral bone tumors. *Clinical Orthopaedics and Related Research*.

[3] O’Connor MI, Sim FH, Chao EYS (1996). Limb salvage for neoplasms of the shoulder girdle: intermediate reconstructive and functional results. *Journal of Bone and Joint Surgery—Series A*.

[4] Getty PJ, Peabody TD (1999). Complications and functional outcomes of reconstruction with an osteoarticular allograft after intra-articular resection of the proximal aspect of the humerus. *Journal of Bone and Joint Surgery—Series A*.

[5] Rödl RW, Gosheger G, Gebert C, Lindner N, Ozaki T, Winkelmann W (2002). Reconstruction of the proximal humerus after wide resection of tumours. *Journal of Bone and Joint Surgery—Series B*.

[6] Yang Q, Li J, Yang Z, Li X, Li Z (2010). Limb sparing surgery for bone tumours of the shoulder girdle: the oncological and functional results. *International Orthopaedics*.

[7] van de Sande MAJ, Dijkstra PD, Taminiau AHM (2011). Proximal humerus reconstruction after tumour resection: biological versus endoprosthetic reconstruction. *International Orthopaedics*.

[8] Abdeen A, Hoang BH, Athanasian EA, Morris CD, Boland PJ, Healey JH (2009). Allograft-prosthesis composite reconstruction of the proximal part of the humerus. Functional outcome and survivorship. *Journal of Bone and Joint Surgery—Series A*.

[9] Black AW, Szabo RM, Titelman RM (2007). Treatment of malignant tumors of the proximal humerus with allograft-prosthesis composite reconstruction. *Journal of Shoulder and Elbow Surgery*.

[10] Ruggieri P, Mavrogenis AF, Guerra G, Mercuri M (2011). Preliminary results after reconstruction of bony defects of the proximal humerus with an allograft-resurfacing composite. *Journal of Bone and Joint Surgery—Series B*.

[11] Kharrazi FD, Busfield BT, Khorshad DS, Hornicek FJ, Mankin HJ (2008). Osteoarticular and total elbow allograft reconstruction with severe bone loss. *Clinical Orthopaedics and Related Research*.

[12] Weber KL, Lin PP, Yasko AW (2003). Complex segmental elbow reconstruction after tumor resection. *Clinical Orthopaedics and Related Research*.

[13] Kocher MS, Gebhardt MC, Mankin HJ (1998). Reconstruction of the distal aspect of the radius with use of an osteoarticular allograft after excision of a skeletal tumor. *Journal of Bone and Joint Surgery—Series A*.

[14] Bianchi G, Donati D, Staals EL, Mercuri M (2005). Osteoarticular allograft reconstruction of the distal radius after bone tumour resection. *Journal of Hand Surgery*.

[15] Szabo RM, Anderson KA, Chen JL (2006). Functional outcome of en bloc excision and osteoarticular allograft replacement with the Sauve-Kapandji procedure for Campanacci grade 3 giant-cell tumor of the distal radius. *Journal of Hand Surgery*.

[16] Enneking WF, Dunham W, Gebhardt MC, Malawar M, Pritchard DJ (1993). A system for the functional evaluation of reconstructive procedures after surgical treatment of tumors of the musculoskeletal system. *Clinical Orthopaedics and Related Research*.

[17] Clavien PA, Barkun J, De Oliveira ML (2009). The clavien-dindo classification of surgical complications: five-year experience. *Annals of Surgery*.

[18] van Isacker T, Barbier O, Traore A, Cornu O, Mazzeo F, Delloye C (2011). Forearm reconstruction with bone allograft following tumor excision: a series of 10 patients with a mean follow-up of 10 years. *Orthopaedics and Traumatology*.

